# Diagnosis of Imported Dengue and Zika Virus Infections in Italy from November 2015 to November 2022: Laboratory Surveillance Data from a National Reference Laboratory

**DOI:** 10.3390/v16010050

**Published:** 2023-12-28

**Authors:** Christina Merakou, Antonello Amendola, Claudia Fortuna, Giulia Marsili, Cristiano Fiorentini, Claudio Argentini, Eleonora Benedetti, Gianni Rezza, Francesco Maraglino, Martina Del Manso, Antonino Bella, Patrizio Pezzotti, Flavia Riccardo, Anna Teresa Palamara, Giulietta Venturi, The Arbovirus Working Group

**Affiliations:** 1 Department of Infectious Diseases, Italian National Institute of Health (ISS), 00161 Rome, Italyantonello.amendola@iss.it (A.A.); claudio.argentini@iss.it (C.A.); antonino.bella@iss.it (A.B.);; 2 ECDC Fellowship Programme, Public Health Microbiology Path (EUPHEM), European Centre for Disease Prevention and Control (ECDC), 16973 Stockholm, Sweden; 3 General Directorate for Health Prevention, Prevention of the Communicable Diseases and International Prophylaxis, Ministry of Health, 00144 Rome, Italy

**Keywords:** Dengue virus, Zika virus, DENV, ZIKV, arboviral infections, arbovirus diagnostics

## Abstract

Dengue (DENV) and Zika (ZIKV) viruses are mosquito-borne human pathogens. In Italy, the presence of the competent vector *Aedes albopictus* increases the risk of autochthonous transmission, and a national plan for arboviruses prevention, surveillance, and response (PNA 2020–2025) is in place. The results of laboratory diagnosis of both viruses by the National Reference Laboratory for arboviruses (NRLA) from November 2015 to November 2022 are presented. Samples from 655 suspected cases were tested by both molecular and serological assays. Virus and antibody kinetics, cross-reactivity, and diagnostic performance of IgM ELISA systems were analysed. Of 524 cases tested for DENV, 146 were classified as confirmed, 7 as probable, while 371 were excluded. Of 619 cases tested for ZIKV, 44 were classified as confirmed, while 492 were excluded. All cases were imported. Overall, 75.3% (110/146) of DENV and 50% (22/44) of ZIKV cases were confirmed through direct virus detection methods. High percentages of cross reactivity were observed between the two viruses. The median lag time from symptoms onset to sample collection was 7 days for both DENV molecular (range 0–20) and NS1 ELISA (range 0–48) tests, with high percentages of positivity also after 7 days (39% and 67%, respectively). For ZIKV, the median lag time was 5 days (range 0–22), with 16% positivity after 7 days. Diagnostic performance was assessed with negative predictive values ranging from 92% to 95% for the anti-DENV systems, and of 97% for the ZIKV one. Lower positive predictive values were seen in the tested population (DENV: 55% to 91%, ZIKV: 50%). DENV and ZIKV diagnosis by molecular test is the gold standard, but sample collection time is a limitation. Serological tests, including Plaque Reduction Neutralization Test, are thus necessary. Co-circulation and cross-reactivity between the two viruses increase diagnostic difficulty. Continuous evaluation of diagnostic strategies is essential to improve laboratory testing.

## 1. Introduction

Dengue (DENV) and Zika (ZIKV) are positive-sense single-stranded RNA viruses, belonging to the genus Orthoflavivirus [1,2] with other important human pathogens such as yellow fever (YFV), West Nile (WNV), Japanese encephalitis (JEV), and tick-borne encephalitis (TBEV) viruses [3,4]. DENV has four genetically distinct serotypes (DENV-1, DENV-2, DENV-3, and DENV-4) [5,6]. DENV and ZIKV are commonly transmitted through the bite of an infected female mosquito of the *Aedes* spp. [7,8]. *Aedes aegypti* and *Aedes albopictus* are both competent vectors [9,10,11], with the latter being present in several European countries, including Italy [12].

Orthoflavivirus-associated human diseases are a growing cause of morbidity and mortality across the world and their area of distribution and co-circulation is expanding. Since 2007, ZIKV outbreaks have been recorded in Africa, the Americas, Asia and the Pacific with a total of 89 countries reporting mosquito-transmitted infections [13]. To date, no autochthonous ZIKV vector-sustained outbreaks have been detected in Italy, however 3 locally-acquired ZIKV disease cases were identified in France in 2019 [14].

DENV is endemic in more than 100 countries with the regions of the Americas, South-East Asia and Western Regions most affected [15]. Overall, more than two billion people live in dengue-endemic regions with a mortality rate due to infection of 5% to 20% [16]. In the mainland of the European Union (EU/EEA), local vectorial transmission of DENV has been documented since 2010 in several countries [17] and in particular in France, where in 2022 65 autochthonous cases were reported over nine separate transmission events, a relevant increase compared to the number of cases observed previously in the country [18]. In Italy, the first autochthonous dengue outbreak was reported in Veneto region in August 2020 [19], and has been recently reported again in Lombardy [20] and Lazio [21]. As of 4 December 2023 [22], over 80 confirmed locally acquired infections were reported in Italy [23]. Currently, given the high burden of DENV outbreaks globally, the likelihood of local transmission events of dengue viruses in EU/EEA areas, where the vector is present, is considered high [21].

Dengue is characterized by a wide spectrum of clinical manifestations. The vast majority of dengue disease cases are asymptomatic or mild and self-managed [15], presenting fever, nausea, vomiting, rash, aches and pains, a positive tourniquet test and leucopenia. Abdominal pain or tenderness, persistent vomiting, clinical fluid accumulation, mucosal bleeding, lethargy, restlessness, and liver enlargement may occur in a small proportion of cases. These symptoms correlate with severe illness, including dengue hemorrhagic fever (DHF) and dengue shock syndrome (DSS), and are characterized by severe plasma leakage leading to shock or fluid accumulation with respiratory distress, severe bleeding, severe organ impairment, impaired consciousness, or cardiovascular disease [24]. A secondary DENV infection (e.g., exposure to a heterotypic serotype) represents the greatest risk factor for serious diseases due to the phenomenon of antibody-dependent enhancement (ADE) [6,24]. Because no specific antiviral therapies exist for treatment, appropriate clinical management through supportive care is the mainstay for reducing morbidity and mortality [25].

In the majority of cases, Zika infection is asymptomatic or associated with mild disease, with the most common symptoms being fever, rash, headache, joint pain, conjunctivitis and muscle pain [26]. Severe manifestations of ZIKV infection are Guillain–Barre syndrome in adults and microcephaly in babies following congenital infection [27].

The changing epidemiology in the EU/EEA of DENV and ZIKV and their often aspecific clinical presentations make the diagnoses, clinical and public health management of fevers among returning travelers, as well as summer fevers in the resident populations, an evolving challenge [28]. Rapid and accurate laboratory diagnosis of suspected DENV/ZIKV imported cases is important for optimal patient management, especially during pregnancy [29,30]. Moreover, it is also a fundamental pillar for arthropod-borne viruses (arbovirus) surveillance in countries in which competent vectors are present, in order to trigger actions to reduce the risk of onward autochthonous transmission.

The Italian response to arboviruses is regulated by a five-year National Plan for Prevention, Surveillance, and Response to Arboviruses (PNA 2020–2025) published by the Ministry of Health. This plan also encompasses the surveillance of arboviruses transmitted by *Aedes* spp., including the Orthoflavivirus DENV and ZIKV. Case definitions are provided for each arbovirus, including laboratory criteria, in agreement with the ECDC case definitions [31,32]. In particular, for a case to be classified as confirmed, laboratory criteria must be met. 

Laboratory tests for DENV and ZIKV infection involve either: directly detecting the virus through viral isolation, molecular detection of the viral genome, or viral NS1 antigen capture for DENV; or indirectly determining the host’s response by measuring antibodies through serological assays, such as enzyme-linked immunosorbent assays (ELISA) (including MAC and GAC-ELISA for the capture of IgM and IgG, respectively), immunofluorescence assays (IF), and Neutralization tests (NT) [33,34,35]. Testing two consecutive serum samples collected at least two weeks apart can help demonstrate seroconversion or an increase in antibodies titers [36]. Given that DENV and ZIKV (and also the Alphavirus Chikungunya-CHIKV) exhibit overlapping clinical presentations and epidemiological distributions, these viruses usually need to be tested in parallel. Direct detection of the virus is usually made by molecular assays, which are fast, highly sensitive and specific, and are sufficient for the confirmation of a suspected case. However, the window of opportunity to detect the viral genome in biological samples is usually limited, and differences in the viral kinetics of the different arboviruses may further hamper detection. Moreover, the continuous genomic evolution of these viruses poses the risk of newly acquired mutations hampering the sensitivity of in-use molecular tests [37]. For serology, cross-reactivity among the different members of the Orthoflavivirus genus complicates diagnostics [38,39]. The detection of neutralizing anti-Orthoflavivirus antibodies by conventional plaque-reduction neutralization test (PRNT) and virus neutralization assay (VNA), is the “gold standard” in discriminant Orthoflavivirus serology. A positive PRNT result is required as a confirmation laboratory criterium in the arboviral case definition. However, serological cross-reactions between Orthoflaviviruses are also reported with this assay. The interpretation of laboratory test results is further complicated in the case of an Orthoflavivirus infection occurring in a patient with previous exposure to a different Orthoflavivirus (i.e., secondary Orthoflavivirus infection or previous vaccination). A previous Orthoflavivirus infection may influence the dynamics of IgM production (possibly produced during secondary infection at lower titers and for a shorter period), viremia, and neutralizing antibodies production (sharp increase in neutralizing antibodies titers, which are broadly cross-reactive against different Orthoflaviviruses). Overall, serological results interpretation can be challenging, and diagnosis can remain inconclusive in the absence of a direct viral detection at early stages of infection.

The evaluation of the performances of existing and new diagnostic assays presents another crucial concern. While many serological assays for Orthoflaviviruses are assessed for their specificity and sensitivity, the aspect of predictive value is often overlooked. Predictive value pertains to the probability of the test accurately providing the correct diagnosis [40]. Positive and negative predictive values (PPV and NPV) are subject to variations according to the prevalence of the disease in a population, and differ between endemic and non-endemic countries, or in epidemic contexts.

In this study, we report the laboratory test results for DENV and ZIKV performed on samples of suspected imported cases at the National Reference Laboratory of Arboviruses (NRLA) of the Istituto Superiore di Sanità (ISS), in the period between November 2015 to November 2022, and the related cases classification. We also report the analysis of viral and antibodies kinetics in our population, and the serological cross-reactivity observed between the two Orthoflaviviruses. Finally, we report the analysis of the performances of the IgM ELISA serological tests in use in our population.

## 2. Materials and Methods

### 2.1. Patients and Samples

The study population included suspected imported DENV/ZIKV cases. Samples from travelers returning in Italy from DENV/ZIKV-endemic countries, referred with suspected DENV or ZIKV infection, and collected between November 2015 and November 2022 were analyzed. Information on age, gender, visited countries, travel dates, and date of onset of symptoms was recorded for each patient in a case-report form. Collected samples were sent to the NRLA at the ISS in Rome, Italy, from national Regional Reference Laboratories for Arboviruses or Hospital Microbiology Laboratories (RRL/HMLs) in agreement with the PNA 2020–2025, for diagnosis and/or for diagnostic confirmation, and/or for cross-evaluation of the different diagnostic methods used in these laboratories. In agreement with PNA 2020–2025 and ECDC laboratory case definition criteria, the following laboratory assays were performed on biological samples of suspected DENV/ZIKV cases: molecular assays, NS1 ELISA (only for DENV), DENV/ZIKV IgM ELISAs, and PRNT. 

### 2.2. Direct Virus Detection Assays

#### 2.2.1. RNA Extraction and Real Time RT-PCRs

Until November 2019, viral RNA was extracted manually from 140 μL of biological sample (serum, whole blood, plasma, urine, cerebrospinal fluid) using the QIAmp viral RNA Mini kit (Qiagen Inc., Valencia, CA, USA), according to manufacturer’s instructions, and stored at −80 °C. Subsequently, viral RNA was extracted from 200 μL of biological sample (serum, whole blood, plasma, cerebrospinal fluid) by means of MagPurix^®®^ Viral/Pathogen Nucleic Acids Extraction Kit A, and from 1000 μL of urine by using MagPurix^®®^ Viral Nucleic Acid Extraction Kit LV on the automated MagPurix 12A platform (ZINEXTS LIFE SCIENCE Corp., New Taipei City, Taiwan), according to manufacturer’s instructions, and stored at −80 °C. RNA was amplified by real time RT-PCR for DENV, and/or ZIKV detection. The primers and probes used in this study can be found in Table S1. All RT-PCR assays were performed by using the SensiFast Probe No-ROX One-Step kit (Bioline Meridian Bioscience, Memphis, TN, USA), according to manufacturer’s protocol, and CFX96 Touch™ Real-Time PCR Detection System (Bio-Rad).

#### 2.2.2. Dengue NS1 Antigen Detection

Dengue virus NS1 antigen was detected in patients’ serum samples using a commercial antigen-capture ELISA system (Bio-Rad Platelia™ Dengue NS1 Ag, Milan, Italy). Absorbance was measured at 450 nm using an ELISA reader (BioTek^®®^ Instruments, Winooski, VT, USA), according to manufacturer’s instructions. Sample optical density (OD) readings were compared with reference cut-off OD readings to determine the result. Ratio values in the range 0.5–1 were considered as borderline (bl) (grey zone); a ratio value ≥ 1 was considered positive for the presence of NS1 antigen. 

### 2.3. Serological Assays

#### 2.3.1. Anti-DENV IgM ELISA Systems

IgM antibodies against DENV were detected in serum samples using the following commercial IgM-capture ELISA systems: 1—Focus Diagnostics Dengue Virus IgM Capture, DxSelect™, (Cypress, CA, USA), herein ELISA IgM system 1; 2—Dia.Pro Dengue Virus IgM (Milan, Italy), herein ELISA IgM system 2; 3—InBios Dengue Detect™ IgM Capture ELISA (FDA) (Seattle, WA, USA), herein ELISA IgM system 3. Absorbance was measured at 450 nm using an ELISA reader, according to manufacturer’s instructions. Sample OD readings were compared with reference cut-off OD readings to determine the result for the presence of IgM antibodies. Ratio values of >1 for system 1 M DENV, >1.1 for system 2 M DENV, and >2.84 for system 3 M DENV, were considered as positive for the presence of anti DENV IgM antibodies. Ratio values in the range 0.9–1.1 for system 2 M DENV and 1.65–2.84 for system 3 M DENV were considered as borderline for the presence of anti DENV IgM antibodies.

#### 2.3.2. Anti-ZIKV IgM ELISA Systems

IgM antibodies against ZIKV were detected in serum samples using the following commercial IgM-capture ELISA systems: 1—Euroimmun Anti-Zika Virus IgM ELISA (Luebeck, Germany), herein ELISA IgM system 4; 2—Dia.Pro Zika Virus IgM (Milan, Italy), herein ELISA IgM system 5. Absorbance was measured at 450 nm using an ELISA reader, according to manufacturer’s instructions. OD readings were compared with reference cut-off OD readings to determine the result for the presence of IgM antibodies. Ratio values of ≥1.1 for both system 1M ZIKV and 2M ZIKV were considered as positive for the presence of anti ZIKV IgM antibodies. Ratio values in the range 0.8–1.1 for system 1M ZIKV and 0.9–1.1 for system 2M ZIKV were considered as bl (grey zone) for the presence of anti ZIKV IgM antibodies.

#### 2.3.3. Plaque Reduction Neutralization Test (PRNT) 

The assay was performed as previously described [41]. Briefly, six-well tissue culture plates with sub-confluent VERO cell monolayer (approximately 70% confluency) were used. The following viruses were used: the DENV-2 (NGB strain) and the ZIKV H/PF/2013 strain [42]. Infectivity titration of each viral strain was performed by PRNT assay using VERO cells. Patients’ sera were diluted 1:10 in serum-free maintenance medium, heat-inactivated, and titrated in duplicate. Equal volumes (100 μL) of DENV/ZIKV dilutions, containing approximately 80 Plaque Forming Units (PFU), and each patient serum dilution were mixed, and incubated overnight at 4 °C. Subsequently, VERO cells plates were infected with 200 μL/well of virus–serum mixtures in duplicate. After 1 h of incubation at 37 °C and 5% CO_2_, the supernatant was aspirated and a 1:1 mixture of 2% Gum Tragacanth and one part of supplemented medium (2× MEM, 2.5% inactivated fetal calf serum (FCS) and 2% 1 M HEPES) was added. The plates were incubated at 37 °C and 5% CO_2_ again, for 4 (ZIKV)/7 (DENV) days, and then stained with 1.5% crystal violet. A titration of DENV/ZIKV with three different dilutions in duplicate (working, 1:2, and 1:8 dilutions) was performed in each assay and used as a control for the assay. Neutralizing antibody titers were calculated as the reciprocal of the serum dilution that gave a 50% or 80% reduction of the number of plaques (PRNT50/PRNT80), as compared to the virus control. PRNT80 ≥ 10 were considered positive, while PRNT50 ≥ 10 were considered as borderline.

### 2.4. Analysis of Results and Statistics 

Based on laboratory test results, suspected cases were classified as confirmed, probable, or were excluded, according to the criteria detailed in Table S2, adapted from those defined by the Italian PNA 2020–2025 [43] and ECDC case definition [31,32]. When samples were tested by more than one IgM ELISA system, as in the case of the same sample resulting positive in one system and negative or borderline in another, the positive value was used for the analysis. Probable IgM-positive cases were further tested by molecular tests and PRNT to be confirmed or excluded. However, they remained classified as probable when a borderline result (PRNT50 ≥ 1:10) was obtained by PRNT, and it was not possible to test a second serum sample collected after at least two weeks. Indeed, in our experience, borderline PRNT results can be obtained early in the course of infection, at the beginning of seroconversion, but they can also be due to cross-reactivity toward a different Orthoflavivirus [41]. Both molecular and serological results, obtained both at the NRLA and at RRL/HMLs, from one or more samples per suspected case, were evaluated for diagnosis and case classification. Laboratory tests were in most cases run in parallel for DENV and ZIKV, and results obtained for both viruses were evaluated for classification. The time-span between symptoms onset and sample collection was also considered, when available. Due to the high cross-reactivity between Orthoflaviviruses, borderline IgM ELISA results were considered as negative in all the analyses, except for the analysis of the IgM antibody kinetics. Of note, we considered as not classified (NC) cases being those not showing IgM-negative results, but PRNT-positive results, since the time of infection could not be established by laboratory data, or cases where laboratory results could not be interpreted due to lacking proper sample type and/or time collection, or due to insufficient information (time of symptoms onset). We also considered as NC for DENV or ZIKV, cases with IgM pos/bl plus PRNT positive/borderline, but confirmed by molecular test for a different Orthoflavivirus (ZIKV or DENV or a different Orthoflavivirus endemic in the area). Moreover, cases for which laboratory results suggested a recent infection by an Orthoflavivirus (detection of IgM antibodies specific for DENV and/or ZIKV) but did not allow for distinction of which Orthoflavivirus, because of the detection of neutralizing antibodies for both DENV and ZIKV, were classified as probable recent Orthoflavivirus infections (PRO) (Table S2).

To evaluate the cross-reactivity of anti-DENV IgM antibodies against ZIKV, DENV confirmed cases (and ZIKV excluded) were included in analysis. For the cross-reactivity of anti-ZIKV IgM antibodies against DENV evaluation, ZIKV confirmed cases (and DENV excluded) were considered. All above defined patients’ tested samples were included in the analysis. For the IgM ELISA systems, only positive results were considered, while for PRNTs, both positive and borderline results were considered.

To evaluate the kinetics of the virus and the antibodies produced against them, the following approach was followed. Positive and borderline results obtained by testing all types of samples from DENV and ZIKV confirmed and probable cases, for which the information of the date of collection and the date of symptoms initiation were available, were included in the analysis. Results from RRL/HMLs were also included in the analysis. Samples were categorized into three time period groups based on the lag time from date of symptoms to date of collection: samples collected within three days from symptom onset (≤3 days), between three to seven days (>3 to ≤7 days) and beyond seven days (>7 days). Data from samples tested in assays that have different systems, such as IgM ELISA and molecular assays, were pooled, as one result. In case that the same sample resulted as positive in one system and negative or borderline in another, the positive value was used for the analysis. The same goes for borderline against negative results.

To evaluate the diagnostic performances of each commercially available anti-DENV/ZIKV IgM ELISA assays, we calculated for each assay the sensitivity, specificity, positive predictive values (PPVs), negative predictive values (NPVs), and 95% confidence interval (95% CI), using the STATA command diagtest. In this analysis, we considered as “disease” (i.e., positive for DENV/ZIKV infection) the cases that were classified as confirmed (samples tested positive at molecular and/or PRNT tests), and as “disease-free” (i.e., negative for DENV/ZIKV infection) the cases that were classified as excluded, on the base of the results of molecular and serological tests according to criteria shown in Table S2. NC cases, probable and PRO cases, as well as confirmed past infections, were excluded from the analyses. When more than one biological sample was available and tested per patient, all the results were included in the analysis. The ELISA results were evaluated according to the manufacturers’ cut-offs values, and bl results were considered as negative for the sensitivity, specificity, PPVs, and NPVs analyses. During the 7-yearperiod covered by the study, new ELISA systems became available on the market and where tested. In some cases, they were used for a short time and substituted with other systems after a period of overlapping use. Moreover, some samples were retrospectively re-tested with different systems. Results obtained with all ELISA systems used in the study period have been analyzed and evaluated.

Data cleaning and data analysis were performed using excel and STATA 16.1. Percentages (%) for symptoms and travel history data were rounded to the whole number, for laboratory tests, viral, and antibody kinetics to the first decimal and for demographics and sensitivity, specificity, and predictive values to the second one.

## 3. Results

### 3.1. Cases Clasification and Laboraotory Tests Results

#### 3.1.1. Cases Classification

Samples collected from 655 patients with a suspected imported arboviral infection were analyzed in the study period. The date of symptoms onset and/or hospitalization was available for 436 subjects and the median lag time before sample collection was 10 days (range 0–372 days, mean ± standard deviation: 19.65 ± 30.09 days). Two serum samples (acute phase and convalescence phase) were available from 16 patients. Samples were sent to the NRLA from several Italian Regions (Abruzzo, Basilicata, Calabria, Campania, Friuli–Venezia Giulia, Lazio, Liguria, Lombardy, Marche, Piedmont, Puglia, Sardinia, Sicily, Trentino-Alto Adige, Tuscany, Umbria, Veneto, 17/20). A total of 36 patients were tested for DENV, 131 for ZIKV and 488 for both. 

Suspected DENV and ZIKV cases were classified as confirmed or probable, or were excluded, based on available laboratory tests results Table 1 (for DENV) and Table 2 (for ZIKV) show the total numbers per year and per case classification. In total, from November 2015 to November 2022, NRLA tested 524 suspected DENV cases, and classified 146 as confirmed, and 7 as probable, while 371 were excluded. Among the 619 ZIKV suspected cases, 44 were classified as confirmed, while 575 were excluded. Co-infection with CHIKV was detected through molecular testing in two cases (one DENV and one ZIKV). A conclusive diagnosis was not possible in 34 (7%) cases of those tested for both DENV and ZIKV: laboratory results suggested a recent infection by an Orthoflavivirus (presence of IgM), but the detection of neutralizing antibodies against both DENV and ZIKV did not allow discrimination and as a result were classified as PRO. Moreover, one case was excluded for ZIKV but classified as PRO because of positive IgM and/or PRNT result for DENV, WNV and Usutu virus. Finally, 48 of 524 (9.2%) cases tested for DENV, and 49 of 619 (7.9%) cases tested for ZIKV could not be classified (NC). Even though laboratory results were consistent with a past DENV/ZIKV/Orthoflavivirus infection, a recent infection could not be excluded, either because of possibly altered IgM kinetics following a secondary Orthoflavivirus infection, or due to possible cross-reactivity. Among NC DENV cases, 10 (20.8%) were confirmed for ZIKV by molecular test, 8 of which showed positive/borderline serological results (IgM positive and PRNT positive or border line for DENV) most probably due to cross-reactivity: these cases would have been classified as confirmed/probable for DENV if ZIKV had not been also tested. Similarly, among NC ZIKV cases, 15 (30.6%) were confirmed for DENV by molecular test and/or antigen NS1 ELISA, 4 of which gave positive serological results (IgM positive and PRNT positive for ZIKV) most probably due to cross-reactivity, which could again allow for them to be to classified as confirmed for ZIKV if DENV had not been tested. Of note, among cases for which a final diagnosis was not obtained for ZIKV, 5 were of pregnant women (2 NC and 3 PRO) requiring ZIKV diagnosis to exclude the risks of congenital infection. 

#### 3.1.2. Demographical, Clinical and Epidemiological Data of Confirmed and Probable Cases

More than half of the confirmed and probable DENV were male (54%, 62/115) and the median age was 36 years (range: 0–82, 135/153). For ZIKV, 47% were male (15/32) and the median age was 37 years (range: 18–68, 34/44). Details on demographics can be found in Table S3.

Data on symptoms of patients was available for 119/153 of the DENV confirmed and probable cases. Almost all patients presented with fever (98%); other most common symptoms were asthenia (87%) and arthralgia (79%). Details on DENV cases symptoms can be found in Figure S2a. For ZIKV, data on symptoms was only available for 31 out of the 44 confirmed cases. Here, the most common symptom was rash (61% of confirmed cases). Other common symptoms were fever (55%) and arthralgia (45%) (Figure S2b). 

Travel information was available for 129 out of 153 DENV confirmed and probable cases. In detail, 59 patients travelled to the South-East Asian Region (SEAR), 32 to the Region of the Americas (AMR), 13 to the Western Pacific Region (WPR), 7 to the African Region (AFR) and 2 to the Eastern Mediterranean Region (EMR), while the remaining 16 patients had visited multiple countries during their travels and were excluded from the analysis. Information on the WHO region and country of infection origin for DENV confirmed and probable cases are shown in Figure S3a,c. For ZIKV patients, travel information was reported for 30 out of the 44 confirmed cases; of which 23 had travelled to AMR, 1 to SEAR, and 6 to more than one country and were thus excluded from this analysis. Details are found in Figure S3b,d.

#### 3.1.3. Laboratory Tests Results

Molecular and serological test results for DENV and ZIKV are shown in Table 3 and Table 4, stratified in confirmed, probable, excluded, NC and PRO cases. Positive results are reported for each assay. We detected DENV genome in 37.9% (36/95) of confirmed tested cases. Of these, molecular positive results were obtained in serum samples for 32 patients (2 of which were also plasma and whole blood (WB) positives), in urine for 1 patient (serum and WB negative), and in WB for 3 patients (1 serum and urine negative, 1 plasma negative, 1 also plasma positive). For ZIKV, we detected the viral genome in 59.1% (13/22) of confirmed tested cases. Of these, molecular positive results were obtained both in serum and in urine samples for 6 patients, in urine but not in serum for 3 patients, in WB for 1 patient, and in serum (no other sample types tested) for 3 patients.

A positive antigen NS1 ELISA result was also obtained in 69.1% of DENV confirmed tested cases (76/110). Interestingly, two patients returning from Thailand in 2017, and presenting with fever, had positive NS1 results and a borderline DENV IgM ELISA test result, but DENV diagnosis could not be confirmed due to lack of seroconversion in the second serum sample obtained 1 month after the acute sample. These two patients, which were tested only for DENV and ZIKV, remained undiagnosed. Overall, 13/314 border line NS1 antigen ELISA results were obtained, of which 5 were in confirmed DENV cases, 2 in probable DENV/PRO cases, and 6 in excluded/NC cases. 

In total, 72 samples from 70 suspected DENV patients were assessed by both molecular test and NS1 ELISA. 22/72 (32.6%) samples tested positive in both assays, 2/72 (2.8%) tested positive for molecular and borderline for NS1 ELISA, 5/72 (6.9%) tested positive for molecular and negative for NS1 ELISA, and 43/72 (59.7%) only tested positive for NS1 ELISA.

Overall, considering the results obtained by both the NRLA and the RRLs/HMLs, as much as 75.3% (110/146) of DENV cases and 50% (22/44) of ZIKV cases were confirmed by direct virus detection methods (molecular tests for ZIKV, and molecular tests and/or NS1 ELISA test for DENV).

Data on the detection of antibodies in response to infection by three ELISA assays and PRNT are also shown in Table 3 and Table 4. Regarding PRNT, overall high proportion of positive results for DENV and ZIKV (or both: 63 samples of 50 cases) were detected among confirmed, NC and PRO cases. In one case, neutralizing antibodies were detected for both ZIKV and for DENV in a serum sample collected soon after birth of a congenital suspected ZIKV case. A serum sample collected a year later tested negative, thus excluding the congenital infection.

Seroconversion was observed in 12 DENV and 4 ZIKV confirmed cases, testing two subsequent serum samples, collected a median of 17 days from each other (range 3–66, mean ± standard deviation: 22.14 ± 13.63) as shown in Table S4.

#### 3.1.4. Cross-Reactivity between DENV and ZIKV

Antibody cross-reactivity between viruses was assessed and results are shown in Table 5 for the cross-reactivity of anti-DENV antibodies of DENV confirmed, ZIKV excluded cases (except past infections) toward ZIKV; and in Table 6 for the cross-reactivity of anti-ZIKV antibodies of ZIKV confirmed, DENV excluded cases (except past infections) toward DENV. High percentages of cross reactivity were observed, particularly by the anti-DENV IgM ELISA systems, but also by PRNTs. 

#### 3.1.5. Viral and Antibody Kinetics of Confirmed and Probable Cases

Information about the lag time between symptoms onset and sample collection was available for 125 samples (113 serum, 6 urine, 2 plasma, and 4 whole blood samples) of 93 confirmed and 4 probable DENV cases (median 7 days, range 0–195 days, mean ± standard deviation: 12.00 ± 18.94 days). The percentages of the positive-borderline samples of the confirmed and probable cases per lag time group for each diagnostic assay can be found in Table 7.

**Figure 1:**
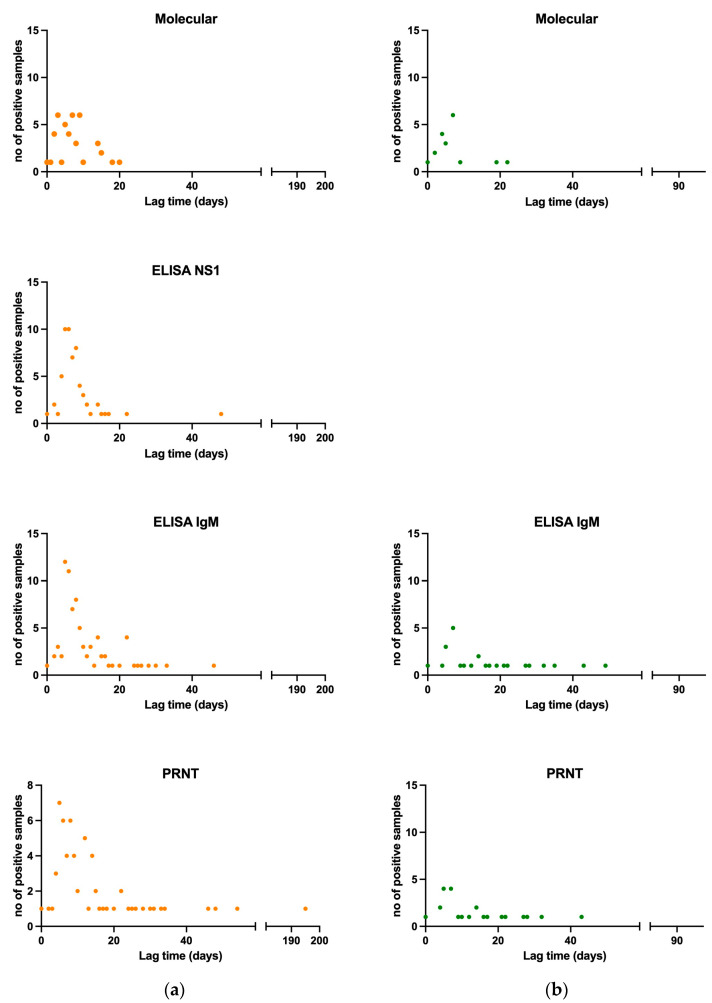
Viral and antibody kinetics per type of sample for molecular and for all samples for all assays tested for DENV confirmed and probable, and for ZIKV confirmed cases. (**a**) Lag time per no of positive samples tested for DENV by molecular, NS1 ELISA, IgM ELISA, and PRNT., in orange (**b**) Lag time per no of positive samples tested for DENV by molecular, NS1 ELISA, IgM ELISA, and PRNT. Lag time is the period between symptom start and sample collection, in green.

Specifically, the median lag time of molecular positive samples (patient *n* = 34) was 7 days (range 0–20, mean ± standard deviation: 6.98 ± 3.23 days). Median lag time of NS1 ELISA positive or borderline samples (patient *n* = 52) was 7 days (range 0–48, mean ± standard deviation: 8.13 ± 6.44 days). As shown, viral genome could be detected in one serum sample as much as 20 days after symptoms onset, and the viral NS1 antigen could be detected in one case as much as 48 days after symptoms onset, with 38.9% and 66.7% of samples positive by molecular and antigen NS1 tests, respectively, after 7 days from symptoms onset. Median lag time of IgM ELISA positive or borderline samples (patient *n* = 80) was 8 days (range 0–49, mean ± standard deviation: 12.04 ± 9.89 days). Median lag time for PRNT positive and border line samples (patient *n* = 85) was 9 days (range 0–372, mean ± standard deviation: 18.38 ± 38.66 days). IgM and neutralizing antibodies were detected in a high proportion of patients (54.5% and 26.7%, respectively) already within the first three days after the onset of symptoms. All DENV molecular and serological tests results per lag time are shown in Figure 1a.

The information about the lag time between symptoms onset and sample collection was available for 56 samples (31 serum, 17 urine, 4 plasma, 2 whole blood samples, and 2 saliva) of 23 confirmed ZIKV cases. Results are shown in Table 8. Median time lag between symptoms onset and sample collection for PCR positive samples (patient *n* = 15) was 5 days (range 0–22, mean ± standard deviation: 6.68 ± 3.35 days). Viral genome could be detected in one urine sample collected as much as 22 days after symptoms onset, and in one plasma sample, collected after 19 days after symptoms onset; a positive molecular test could be obtained in 15.8% of samples collected after 7 days from symptoms onset. Median time lag between symptoms onset and sample collection for IgM ELISA positive and borderline samples (patient *n* = 21) was 12 days (range 0–67, mean ± standard deviation: 16.94 ± 15.27 days). Median time lag between symptoms onset and sample collection for PRNT positive and borderline samples (patient *n* = 22) was 8 days (range 0–49, mean ± standard deviation: 14.81 ± 12.37 days). Despite the low number of samples (*n* = 3) analyzed within 3 days from symptom onset, IgM and neutralizing antibodies were detected in a high proportion of patients (33% and 100%, respectively). All ZIKV molecular and serological tests results per lag time are shown in Figure 1b.

A correlation between lag time, type of sample and type of assay was attempted but the number of samples was too limited to conclude for both DENV and ZIKV samples. Nonetheless, for the molecular tests a heat map of sample origin and lag timing can be found in Figure S4a,b.

#### 3.1.6. Diagnostic Performance Assessment of Commercially Available ELISA Systems

Results of sensitivity, specificity, and predictive values of 4 IgM ELISA systems (system 1 M, 2 M, and 3 M for DENV, and system 2 M for ZIKV) are reported in Table 9. The number of “confirmed” and “excluded” case samples tested with each of the ELISA system is also shown. Overall, sensitivity values ranging from 81.5% to 88.66% and specificity values ranging from 79.07% and 95.26% were observed. While we observed negative predictive values (NPVs) ranging from 91.78% to 94.44% for DENV, and of 97.19% for ZIKV, lower positive predictive values (PPVs) were seen, ranging from 55% to 90.53% for DENV, and 50% for ZIKV.

## 4. Discussion

In the past decade, different arboviruses, such as DENV (different serotypes), ZIKV, and CHIKV have dramatically spread in previously unaffected areas worldwide, where they now co-circulate and cause recurrent outbreaks [18,19,20,44,45,46]. Concurrently, their competent vector species are also spreading to non-tropical areas, where autochthonous transmission events are increasingly being reported [18,19,20]. In this context of a continuously evolving epidemiological situation, arboviruses surveillance is of the utmost importance for the implementation of prevention and control measures. Due to overlapping epidemiological characteristics and clinical presentations, the laboratory investigation of arboviruses is essential for differential diagnosis, as well as for case classification for surveillance purposes. Confirmation of DENV and ZIKV suspected cases requires, as per the European and the Italian case definition, both direct viral detection assays (molecular tests, viral antigen detection, viral isolation) and/or serological tests (detection of specific IgM antibodies, indicating recent infection, confirmed by neutralization tests, and/or seroconversion).

Our analysis focused on the laboratory investigation of over 650 samples for the diagnosis of the Orthoflavivirus DENV and ZIKV, performed at the Italian NRLA, between November 2015 and November 2022. While most of the samples were also tested for the Alphavirus CHIKV, in this study we focused on the related Orthoflaviviruses, ZIKV and DENV, whose differential diagnosis still represents a challenge. The study period covers the 2016 ZIKV epidemic peak in Latin America and the related peak of imported cases in Italy. Even if ZIKV had already caused outbreaks starting from 2007 in the remote Yap Island (Micronesia), and then in 2013–2014 in several Pacific Ocean Islands, diagnosis began to be a serious public health challenge after its emergence in the Americas. In Italy, the first ZIKV imported case was detected in a traveler returning to Europe from Brazil in March 2015 [47]. 

The demand for ZIKV diagnosis sharply increased when the virus was associated with microcephaly [48]. During the first phase of the epidemic, reference laboratories faced a growing demand for diagnosis with only in-house developed molecular and serological tests at their disposal, and no reference material and validated assays available. Moreover, in non-endemic countries, biological samples from disease cases, which are essential for the evaluation and validation of diagnostic systems, were lacking. ZIKV emergence, coupled with increasing co-circulation of multiple DENV serotypes, has made Orthoflavivirus diagnosis, particularly the serological diagnosis, a challenge. Importantly, the request for ZIKV diagnosis was often prompted not by the presence of symptoms, but rather by the necessity to rule out the risk of infection during pregnancy in women returning from epidemic areas. This condition further complicated the interpretation of laboratory results, given the lack of time for infection estimates. 

In our study, most of the ZIKV cases were detected in 2016, linked to the epidemic in Brazil, while DENV cases were detected every year except for 2020 and 2021 when traveling was limited due to the COVID-19 pandemic. We have also observed a sharp decrease in the number of biological samples received by NRLA after the end of the pandemic, possibly because of increased laboratory testing capacity. However, DENV imported cases were again detected in 2022, with the resumption of traveling, an observation in agreement with the National epidemiological surveillance data, which also includes cases detected by RRLs [49]. 

Our results confirm the importance of genome and/or antigen direct detection for diagnosis and case confirmation, and highlight the importance of proper and timely collection and testing of different biological sample types. DENV NS1 antigen detection by an ELISA system has been shown to increase the time window for direct DENV detection. Based on viral and antibody kinetics analysis, testing for direct pathogen identification should be attempted also in biological samples collected after 7 days following symptoms onset. Furthermore, serological testing should also be performed in sera collected within 3 days from symptoms onset. Our study also shows that, although in a limited percentage of suspected cases, available diagnostic assays and laboratory criteria for case classification are not sufficient for a final diagnosis, with cross-reactivity between closely related Orthoflaviviruses being the main limitation in serological testing. A crucial takeaway message from our findings is that in some instances, cases could be erroneously classified as confirmed for DENV or ZIKV when applying the available case definition criteria if not tested in parallel for both viruses, or for all Orthoflavivirus known to circulate in a certain area. In Italy, there is a prevalent presence of various Orthoflaviviruses of concern, including West Nile, Usutu, and Tick-Borne Encephalitis viruses [49].

Finally, the evaluation of commercially available serological systems used in our study has shown important differences in the diagnostic performances of the different systems, highlighting the importance of such evaluation in different contexts and evolving scenarios.

A better understanding by clinicians of the difficulties of DENV and ZIKV differential diagnosis will help improve the accuracy and completeness of sample type collection and necessary information to correctly perform the diagnosis and interpret the results. Furthermore, new laboratory diagnostic approaches are needed, and will need to be evaluated. This need for evaluation brings to light another problem, that of the lack of biological sample collections of disease cases. These collections are rare to find, especially in non-endemic countries. This issue can only be addressed through joint efforts and networking. 

## 5. Conclusions

Confirmation of DENV and ZIKV suspected cases requires, as per ECDC and Italian PNA 2020–2025 case definition, the execution of both direct viral detection assays and serological tests. Limitations of diagnostic tests performed to directly detect the virus include limited and variable time of virus presence in different biological sample types, while for serology the main issue is cross-reactivity. The increasing frequency of secondary infections due to the evolving epidemiological situation at the global level further hampers the interpretation of laboratory results. Our results show that the available diagnostic assays and laboratory criteria for case classification are not sufficient for a final diagnosis in a limited percentage of suspected cases. Moreover, better understanding by clinicians of the difficulties of DENV and ZIKV differential diagnosis due to lack of necessary background information and/or different sample types’ collection, will help towards a correct diagnosis and interpretation of results. 

## Figures and Tables

**Table 1:** NRLA DENV laboratory tested cases per year and per case classification from November 2015 to November 2022. Patients with past infections are indicated. NC: Not Classified, PRO: Probable Recent Orthoflavivirus.

	NRLA Cases Classification for DENV					
Year	Confirmed	Probable	Excluded	NC	PRO	Total
Nov-2015	9 ^1^	0	9	1	2	21
2016	46 ^1^	5	96	19	11	177
2017	29	1	46	9	7	92
2018	17 ^1^	0	57	7	3	84
2019	38 ^1^	1	60	10	10	119
2020	0	0	10	0	1	11
2021	0	0	6	0	0	6
Nov-2022	8	0	4	1	1	14
Total	146	7	288	48	35	524

^1^ Out of 146 confirmed cases, 4 were retrospectively diagnosed, 1 per year in the years 2015, 2016, 2018, 2019.

**Table 2:** NRLA ZIKV laboratory tested cases per year and per case classification from November 2015 to November 2022. Patients with past infections are indicated. NC: Not Classified, PRO: Probable Recent Orthoflavivirus.

	NRLA Cases Classification for ZIKV					
Year	Confirmed	Probable	Excluded	NC	PRO	Total
Nov-2015	0	NA	14	2	2	18
2016	32	NA	212	17	11	272
2017	8 ^1^	NA	68	8	7	91
2018	1	NA	72	9	3	85
2019	3	NA	94	9	10	116
2020	0	NA	10	1	0	11
2021	0	NA	11	0	0	11
Nov-2022	0	NA	11	3	1	15
Total	44	NA	492	49	34	619

^1^ Out of 8 confirmed infections diagnosed in 2017, 4 were retrospectively diagnosed.

**Table 3:** Diagnostic laboratory tests results for DENV from 524 patients that were visited/hospitalized with a suspected DENV infection. Total, confirmed, probable, excluded, not classified (NC) and probable recent Orthoflavivirus (NRO) cases are reported. One sample per patient per diagnostic method was included in the analysis. Percentages are calculated considering positive divided by total tested and are rounded to the first decimal. For PRNT assay borderline values are also reported but not included in the % calculations.

DENV	Tot ^a^ Patients (N = 524)	Confirmed (N = 146)	Probable (N = 7)	Excluded (N = 288)	NC (N = 48)	PRO (N = 35)
Molecular test pos ^b^/tested (%)	36/318 (11.3%)	36/95 (37.9%)	0/4 (0.0%)	0/173 (0.0%)	0/26 (0.0%)	0/20 (0.0%)
NS1 antigen ELISA pos/tested (%)	78/311 (25.1%)	76/110 (69.1%)	0/5 (0.0%)	2/124 (1.6%)	0/44 (0.0%)	0/28 (0.0%)
ELISA IgM system 1 pos/tested (%)	38/121 (31.4%)	26/29 (89.7%)	2/3 (66.7%)	4/67 (6.0%)	1/12 (8.3%)	5/10 (50.0%)
ELISA IgM system 2 pos/tested (%)	27/65 (41.5%)	11/14 (78.6%)	1/2 (50.0%)	7/37 (18.9%)	4/7 (57.1%)	4/5 (80.0%)
ELISA IgM system 3 pos/tested (%)	103/358 (28.8%)	79/101 (78.2%)	3/5 (60.0%)	6/181 (3.3%)	5/43 (11.6%)	10/28 (35.7%)
PRNT pos + bl ^c^/tested (%)	168 + 59/508 (33.1%)	99 + 30/145 (68.3%)	0 + 7/7 (0.0%)	1 + 9/273 (0.4%)	37 + 9/48 (77.1%)	31 + 4/35 (88.6%)

^a^ tot: total. ^b^ pos: positive. ^c^ bl: borderline.

**Table 4:** Diagnostic laboratory tests results for ZIKV from 619 patients that visited/were hospitalized with a suspected ZIKV infection. Total, confirmed, probable, excluded, not classified (NC), and probable recent Orthoflavivirus (PRO) cases are reported. One sample per patient per diagnostic method was included in the analysis. Percentages are calculated considering positive divided by total tested and are rounded to the first decimal. For PRNT assay borderline values are also reported but not included in the % calculations.

ZIKV	Tot ^a^ Patients (N = 619)	Confirmed (N = 44)	Probable (N = 0)	Excluded (N = 492)	NC (N = 49)	PRO (N = 34)
Molecular test pos ^b^/tested	13/411 (3.1%)	13/22 (59.1%)	NA	0/345 (0.0%)	0/25 (0.0%)	0/19 (0.0%)
ELISA IgM system 4 pos/tested	5/30 (16.7%)	4/7 (57.1%)	NA	0/11 (0.0%)	0/9 (0.0%)	1/3 (33.3%)
ELISA IgM system 5 pos/tested	104/487 (21.4%)	34/44 (77.3%)	NA	41/363 (11.3%)	12/48 (25.0%)	17/32 (53.1%)
PRNT pos + bl ^c^/tested	95 + 54/594 (16%)	38 + 5/44 (86%)	NA	1 + 28/466 (0.2%)	29 + 14/49 (59%)	27 + 7/34 (79%)

^a^ tot: total. ^b^ pos: positive. ^c^ bl: borderline. NA = Non applicable.

**Table 5:** Cross-reactivity of anti-DENV antibodies against two ZIKV IgM ELISA systems and PRNT. Only DENV confirmed cases that are also ZIKV excluded are taken into consideration. All tested samples per patient are included in the analysis. For IgM ELISA systems % are calculated by positive to tested samples and for PRNT assays adding positive and borderline results divided by tested samples.

	Anti-ZIKV IgM ELISA System 5			Anti-ZIKV PRNT			
DENV confirmed (*n* = 98)	pos ^a^	tested ^c^	%	pos	bl ^b^	tested	%
	10	106	9.4	0	31	108	28.7

*n*: number of patients, ^a^ pos: positive; ^b^ bl: borderline; ^c^ tested: all samples tested even from same patient, %: pos plus bl/total for PRNT.

**Table 6:** Cross-reactivity of anti-ZIKV antibodies against three DENV IgM ELISA systems and PRNT. Only ZIKV confirmed cases that are also DENV excluded are taken into consideration. All tested samples per patient are included in the analysis. For IgM ELISA systems % are calculated by positive to tested samples and for PRNT assays adding positive and borderline results divided by tested samples.

	Anti-DENV IgM ELISA System 1			Anti-DENV IgM ELISA System 2			Anti-DENV IgM ELISA System 3			Anti-DENV PRNT			
ZIKV confirmed (*n* = 30)	pos ^a^	tested ^c^	%	pos	tested	%	pos	tested	%	pos	bl ^b^	tested	%
	3	12	25.0	9	29	45.0	7	29	24.1	0	6	39	15.4

*n*: number of patients, ^a^ pos: positive; ^b^ bl: borderline; ^c^ tested: all samples tested even from same patient, %: pos plus bl/total for PRNT.

**Table 7:** Viral and antibody kinetics for DENV. DENV confirmed and probable cases viral and antibody kinetics of all samples tested for molecular, NS1 ELISA, IgM ELISA, and PRNT. Lag time from symptom start to sample collection is divided in three groups of ≤3 days, >3 to 7 days, and >7 days. % are positive plus borderline divided by total samples tested, apart from molecular which is only positive divided by tested. Results from RRL/HML are included in the analysis for molecular and NS1 ELISA test.

DENV	≤3 Days				>3 to 7 Days				>7 Days			
	pos	b.l.	Total	%	pos	b.l.	Total	%	pos	b.l.	Total	%
Molecular	11	NA	14	78.6	15	NA	37	40.5	14	NA	36	38.9
NS1 ELISA	3	0	5	60.0	30	1	35	88.6	22	4	39	66.7
IgM ELISA	6	0	11	54.5	31	6	44	84.1	41	2	49	87.8
PRNT	2	2	15	26.7	20	15	44	79.5	38	12	52	96.2

**Table 8:** Viral and antibody kinetics for ZIKV. ZIKV confirmed cases viral and antibody kinetics of all samples tested for molecular, IgM ELISA and PRNT. Lag time from symptom start to sample collection is divided in three groups of ≤3 days, >3 to 7 days, and >7 days. % are positive plus borderline divided by total samples tested apart from molecular which is only positive divided by tested. Results from RRL/HML are included in the analysis for molecular test.

ZIKV	≤3 Days				>3 to ≤7 Days				>7 Days			
	pos	b.l.	Total	%	pos	b.l.	Total	%	pos	b.l.	Total	%
Molecular	3	NA	4	75.0	15	NA	23	65.2	3	NA	19	15.8
IgM ELISA	1	0	3	33.3	9	3	14	85.7	16	0	16	100.0
PRNT	1	2	3	100.0	10	2	14	85.7	13	1	14	100.0

**Table 9:** Sensitivity, specificity, and predictive values (95% CI) for DENV in IgM ELISA systems 1, 2, 3 and ZIKV in IgM ELISA system 5.

DENV		Confirmed (*n* = 29)	Excluded (*n* = 66)	Total
IgM ELISA system 1	Positive	30	4	34
	Negative	6	67	73
	Total	36	71	107
	Value (95% CI)			
Sensitivity	83.33% (76.27, 90.39)			
Specificity	94.37% (90.00, 98.74)			
PPV	88.24% (82.13, 94.34)			
NPV	91.78% (86.58, 96.98)			
		Confirmed (*n* = 13)	Excluded (*n* = 37)	Total
IgM ELISA system 2	Positive	11	9	20
	Negative	2	34	36
	Total	13	43	56
	Value (95% CI)			
Sensitivity	84.62% (75.17, 94.07)			
Specificity	79.07% (68.41, 89.72)			
PPV	55.00% (41.97, 68.03)			
NPV	94.44% (88.45, 100.44)			
DENV		Confirmed (*n* = 85)	Excluded (*n* = 156)	Total
IgM ELISA system 3	Positive	86	9	95
	Negative	11	181	192
	Total	97	190	287
	Value (95% CI)			
Sensitivity	88.66% (84.99, 92.33)			
Specificity	95.26% (92.81, 97.71)			
PPV	90.53% (87.15, 93.90)			
NPV	94.27% (91.58, 96.96)			
Notes: 9 of DENV confirmed cases showed a positive molecular but negative IgM ELISA results (4 for system 1, 1 for system 2 and 4 for system 4).				
ZIKV		Confirmed (*n* = 37)	Excluded (*n* = 344)	Total
IgM ELISA system 5	Positive	44	44	88
	Negative	10	346	356
	Total	54	390	448
	Value (95% CI)			
Sensitivity	81.48% (77.87, 85.09)			
Specificity	88.72% (85.78, 91.65)			
PPV	50.00% (45.35, 54.65)			
NPV	97.19% (95.65, 98.73)			
Notes: 4 of ZIKV confirmed cases showed a positive molecular result, but negative IgM ELISA results for system 5.				

## Data Availability

Data is contained within the article and Supplementary Materials.

## Supplementary Materials

The following supporting information can be downloaded at: https://www.mdpi.com/article/10.3390/v16010050/s1. Figure S1: Flow chart of the criteria for the interpretation of laboratory tests results for Orthoflavivirus diagnosis (ZIKV/DENV) and case classifications. Figure S2: Symptomatology of DENV and ZIKV by % of confirmed and probable cases that data were available. a. Clinical symptoms of DENV confirmed and probable cases. b. Clinical symptoms of ZIKV confirmed cases. Figure S3: Percentages of DENV and ZIKV confirmed and probable cases by WHO region and by country visited before or during clinical symptoms presentation that data were available. Cases that had visited more than one country are not included in the graphs. a. Percentages of DENV confirmed and probable cases by WHO region. b. Percentages of ZIKV confirmed cases by WHO region. c. Percentages of DENV confirmed and probable cases by country. b. Percentages of ZIKV confirmed cases by country. Figure S4: Heat map of sample type, no. of samples per type and on lag time days for a. DENV and b. ZIKV that were tested by molecular assay. Table S1: Molecular tests used for the diagnosis of DENV/ZIKV infection. The genus species group, the target gene, the type of the oligo, the oligo IS, the 5′-3′ sequence, the expected PCR fragment in bp and references are provided. Table S2: Criteria for the interpretation of laboratory tests results for Flavivirus diagnosis (ZIKV/DENV) and case classifications. Table S3: DENV and ZIKV confirmed and probable cases’ gender and age information. No (%) of cases with this information available, no (%) for each gender and age median [range] and mean (±SD) in years are provided. Table S4: Seroconversion data of confirmed cases for DENV and ZIKV. a. DENV patients that showed seroconversion. b. DENV patients that showed seroconversion. Assay type, first and consecutive result, first and second sample lag time and period for seroconversion are showed; The Arbovirus Working Group. References [50,51] are cited in supplementary file.
