# Supplementary material for: Diagnosis of Imported Dengue and Zika Virus Infections in Italy from November 2015 to November 2022: Laboratory Surveillance Data from a National Reference Laboratory
Source: Viruses. 2023 Dec 28;16(1):50. doi: 10.3390/v16010050 (PMC10818496; doi:10.3390/v16010050)
Supplement: Supplementary file 1 [file viruses-16-00050-s001.zip › viruses-2699760-supplementary.pdf]

The Arbovirus Working Group Member: Guido Di Donato [4], Federica Monaco [4], Chiara Pinoni [4], Fabrizia Valleriani [4], Fabio Barbone [5,6], Ludovica Segat [5], Maria Rita Gismondo [7,8], Alessandro Mancon [7], Davide Mileto [7], Elisa Burdino [9], Valeria Ghisetti [9], Maria Grazia Milia [9], Ferdinando Coghe [10], Cinzia Calà [11], Simona De Grazia [11], Alessandro Bartoloni [12,13,14], Maria Grazia Colao [13], Lorenzo Zammarchi [12,13,14], Filippo Lagi [12], Simona Pollini [12,13], Michele Spinicci [12,13,14], Mauro Pistello [15], Elisabetta Giacobazzi [16], Elisabetta Pagani [16], Veronica Bizzotti [1], Alessia Cararelli [1], Valentina Cecchetti [1]

1 Italian National Institute of Health (ISS), Department of Infectious Diseases, Rome, Italy; Vveronica.bizzotti@iss.it (V.B.); alessia.caratelli@iss.it (A.C.); valentina.cecchetti@iss.it (V.C.)
2 ECDC fellowship Programme, Public Health Microbiology path (EUPHEM), European Centre for Disease Prevention and Control (ECDC), Stockholm, Sweden
3 General Directorate for Health Prevention, Prevention of the Communicable Diseases and International Prophylaxis, Ministry of Health, Italy
4 Istituto Zooprofilattico Sperimentale dell'Abruzzo e del Molise "G. Caporale", Teramo, 64100 Italy; gu.didonato@izs.it (G.D.D.); f.monaco@izs.it (F.M.); c.pinoni@izs.it (C.P.); f.valleriani@izs.it (F.V.)
5 Hygiene and Preventive Medicine Clinical Unit, Trieste University Hospital (ASUGI), Trieste, 34128 Italy; fabio.barbone@regione.fvg.it (F.B.); ludovica.segat@asugi.sanita.fvg.it (L.S.)
6 Department of Medical, Surgical and Health Sciences, University of Trieste, Trieste, 34127Italy
7 Laboratory of Clinical Microbiology, Virology and Bioemergencies, "L. Sacco" University Hospital, Milan, 20157 Italy; mariarita.gismondo@unimi.it (M.R.G.); mancon.alessandro@asst-fbf-sacco.it (A.M.); mileto.davide@asst-fbf-sacco.it (D.M.)
8 Department of Biomedical and Clinical Sciences "L. Sacco", University of Milan, Milan, 20157 Italy
9 Department of Infectious Diseases, Amedeo di Savoia Hospital-ASL Città di Torino, Torino, 10149 Italy; elisa.burdino@aslcittaditorino.it (E.B.); valeria.ghisetti@aslcittaditorino.it (V.G.); mariagrazia.milia@aslcittaditorino.it (M.G.M.)
10 Clinical-Microbiological Laboratory, University Hospital of Cagliari, Cagliari, 09127 Italy; fcoghe@aoucagliari.it
11 Microbiology and Virology Complex Operative Unit, University Hospital "P. Giaccone", Palermo, 90127 Italy; cinzia.cala@unipa.it (C.C.); simona.degrazia@unipa.it (S.D.G.)
12 Department of Experimental & Clinical Medicine, University of Florence, Florence, 50121 Italy; alessandro.bartoloni@unifi.it (A.B.); lorenzo.zammarchi@unifi.it (L.Z.); lagif@aou-careggi.toscana.it (F.L.); simona.pollini@unifi.it (S.P.); michele.spinicci@unifi.it (M.S.)
13 Infectious and Tropical Diseases Unit, Careggi University Hospital, Florence, 50134 Italy; colaog@aou-careggi.toscana.it
14 Tuscany Regional Referral Center for Tropical Diseases, Florence, 50134 Italy
15 Department of Translational Research, University of Pisa, Pisa, 56126 Italy; mauro.pistello@unipi.it
16 Lehr-Krankenhaus der Paracelsus Medizinischen Privatuniversität (PMU) Laboratory of Microbiology and Virology, Hospital of Bolzano (SABES-ASDAA), Bolzano-Bozen, 39100 Italy; elisabetta.giacobazzi@sabes.it (E.G.); elisabetta.pagani@sabes.it (E.P.)

Table S1: Molecular tests used for the diagnosis of DENV/ZIKV infection. The genus species group, the target gene, the type of the oligo, the oligo IS, the 5'-3' sequence, the expected PCR fragment in bp and references are provided.

Genus species group	Target gene	Oligo type	Oligo ID	Sequence (5'-3')	PCR fragment (bp)	Reference
DENV	3' noncoding region	fwd[a]	DenS	GGATAGACCAGAGATCCTGCTGT	79	
		rev[b]	DenAs	CATTCCATTTTCTGGCGTTC		
		rev	DenAs1	CAATCCATCTTGCGGCGCTC		
		probe	DenP	FAM-CAGCATCATTCCAGGCACAG-TAMRA		[50]
ZIKV	envelope E protein	fwd	Zvf1086	CCGCTGCCCAACACAAG	53	
		rev	ZIKV 1162c	CCACTAACGTTCTTTTGCAGACAT		
		probe	ZIKV 1107-FAM	FAM-AGCCTACCTTGACAAGCAGTCAGACACTCAA-TAMRA		[51]
Trioplex	DENV 5'UTR	fwd	DENV-F	TAGTCTRCGTGGACCGACAAG		

(DENV, ZIKV, CHIKV)						
		rev	DENV-R1	CAGTTGACACRCGGTTTCTC		
		rev	DENV-R2	GGGTTGATACGCGGTTTCTC		
		probe	DENV-P	FAM-CGYCTWTCAATATGCTGAAACGCG-BHQ1		
	CHIKV nsp1	fwd	CHIKV-F	ACCATCGGTGTTCCATCTAAAG		
		rev	CHIKV-R	GCCTGGGCTCATCGTTATT		
		probe	CHIKV-P	HEX-ACAGTGGTTTCGTGTGAGGGCTAC-BHQ1		
	ZIKV env gene	fwd	ZIKV-F	CCGCTGCCCAACACAAG		
		rev	ZIKV-R	CCACTAACGTTCTTTTGCAGACAT		
		probe	ZIKV-P	TexasRed-AGCCTACCTTGACAAGCAGTCAGACACTCAA-BHQ2		CDC Trioplex Real-time RT-PCR Assay

[a] fwd: forward, [b] rev: reverse

Table S2: Criteria for the interpretation of laboratory tests results for Orthoflavivirus diagnosis (ZIKV/DENV) and case classifications

	**ZIKV**	**DENV**	**Orthoflavivirus**
**Laboratory tests results for probable case**	ZIKV IgM ELISA positive<br>**plus**<br>ZIKV PRNT borderline (case no classified as probable or confirmed for DENV or other Orthoflaviviruses endemic to the region where exposure occurred)	DENV IgM ELISA positive<br>**plus**<br>DENV PRNT borderline (case no classified as probable or confirmed for ZIKV or other Orthoflaviviruses endemic to the region where exposure occurred)	- ZIKV and/or DENV IgM ELISA positive<br>**plus**<br>PRNT positive/borderline for ZIKV **and** DENV (or other Orthoflaviviruses endemic to the region where exposure occurred): **Probable Recent Orthoflavivirus (PRO)**
**Laboratory tests results for confirmed case**	- Culture of ZIKV from biological sample; **OR**<br>- Detection of ZIKV viral RNA in biological samples [serum, urine, cerebrospinal fluid, placenta, umbilical cord, fetal tissue, or other specimen (e.g., amniotic fluid, semen, saliva)]; **OR**<br>- ZIKV IgM ELISA positive<br>**plus**<br>ZIKV PRNT positive (case not classified as probable or confirmed for DENV or other Orthoflaviviruses endemic to the region where exposure occurred); OR<br>- Seroconversion or four-fold antibody titer increase of ZIKV - specific antibodies in paired serum samples<br>**plus**<br>ZIKV PRNT positive (case not classified as probable or confirmed for DENV or other Orthoflaviviruses endemic to the region where exposure occurred)	- Culture of DENV from biological sample; **OR**<br>- Detection of DENV antigen or viral RNA in biological samples; **OR**<br>- DENV IgM ELISA positive<br>**plus**<br>DENV PRNT positive (case no classified as probable or confirmed for ZIKV or other Orthoflaviviruses endemic to the region where exposure occurred); **OR**<br>- Seroconversion or four-fold antibody titer increase of DENV - specific antibodies in paired serum samples<br>**plus**<br>DENV PRNT positive (case no classified as probable or confirmed for ZIKV or other Orthoflaviviruses endemic to the region where exposure occurred)	
**Laboratory tests results for excluded infection**	- ZIKV IgM ELISA positive/borderline, not confirmed by PRNT, nor by molecular test<br>- ZIKV PRNT positive/borderline without an increase in the titer in two consecutive samples;<br>- ZIKV IgM ELISA negative/borderline and ZIKV PRNT borderline in a patient with a confirmed, PRNT positive, recent infection by a different Orthoflavivirus	- DENV IgM ELISA positive/borderline not confirmed by PRNT, nor by molecular test;<br>- DENV PRNT positive/borderline without an increase in the titer in two consecutive samples;<br>- DENV IgM ELISA negative/borderline and DENV PRNT borderline in a patient with a confirmed, PRNT positive, recent infection by a different Orthoflavivirus	
Laboratory tests results for **Not Classified** case (possible past/recent DENV/ZIKV/Orthoflavivirus infection)	- ZIKV IgM ELISA positive/borderline plus ZIKV PRNT bl in late, convalescent samples<br>- ZIKV IgM positive/borderline plus ZIKV PRNT positive/borderline in a patient with a confirmed (molecular test	- DENV IgM ELISA positive/borderline plus DENV PRNT borderline in late, convalescent samples<br>- DENV IgM positive/borderline plus DENV PRNT positive/borderline in a patient with a confirmed (molecular test	- ZIKV and/or DENV IgM ELISA negative/borderline, plus PRNT positive/borderline for ZIKV and/or DENV (or other Orthoflaviviruses endemic to the region where exposure occurred)

	positive) recent infection by a different Orthoflavivirus<br>- ZIKV IgM ELISA negative/borderline, plus PRNT positive	positive) recent infection by a different Orthoflavivirus<br>- DENV IgM ELISA negative/borderline, plus PRNT positive	

Table S3: *DENV and ZIKV confirmed and probable cases' gender and age information. No (%) of cases with this information available, no (%) for each gender and age median [range] and mean (± SD) in years are provided.*

			**Total (%)**
**DENV confirmed and probable cases**			**153 (100,00)**
**Gender**			115 (75.16)
	Male (%)	62 (53.91)	
	Female (%)	53 (46.09)	
**Age (in years)**			148 (95.48)
	Median [range]	36 [0-82]	
	Mean (± SD)	36,91 (±14,72)	
**ZIKV confirmed cases**			**44 (100,00)**
**Gender**			32 (72.72)
	Male (%)	15 (46.88)	
	Female (%)	17 (53.13)	
**Age (in years)**			45 (100,00)
	Median [range]	37 [18-68]	
	Mean (± SD)	39, 07 (±10.10)	

Table S4: Seroconversion data of confirmed cases for DENV and ZIKV. a. DENV patients that showed seroconversion. b. DENV patients that showed seroconversion. Assay type, first and consecutive result, first and second sample lag time and period for seroconversion are showed.

a.

DENV						
**Patient**	**Assay type**	**First result**	**Second result**	**First sample lag time (days)**	**Second sample lag time (days)**	**Seroconversion period (days)**
**1**	PRNT	neg	pos	NA	NA	57
**2**	IgM ELISA	neg	pos	3	22	19
	PRNT	neg	b.l.			
**3**	PRNT	b.l.	pos	5	26	21
**4**	PRNT	b.l.	pos	NA	NA	NA
**5**	IgM ELISA	neg	pos	3	6	3
	PRNT	neg	b.l.			
**6**	IgM ELISA	pos	neg	NA	NA	30
**7**	PRNT	b.l.	pos	4	25	21
**8**	IgM ELISA	b.l.	pos	NA	NA	NA
**9**	PRNT	neg	pos	1	12	11
**10**	PRNT	neg	b.l.	6	21	15
**11**	PRNT	neg	pos	2	15	13
**12**	PRNT	neg	pos	NA	NA	31

Symptom start is counted as day 0.

b.

ZIKV						
**Patient**	**Assay type**	**First result**	**Second result**	**First sample lag time (days)**	**Second sample lag time (days)**	**Seroconversion period (days)**
**1**	PRNT	b.l.	pos	7	14	7
**2**	PRNT	b.l.	pos	NA	NA	66
**3**	PRNT	b.l.	Pos	19	22	3
**4**	IgM ELISA	neg	pos	3	17	13
	PRNT	b.l.	pos			

Symptom start is counted as day 0.

Figure S1

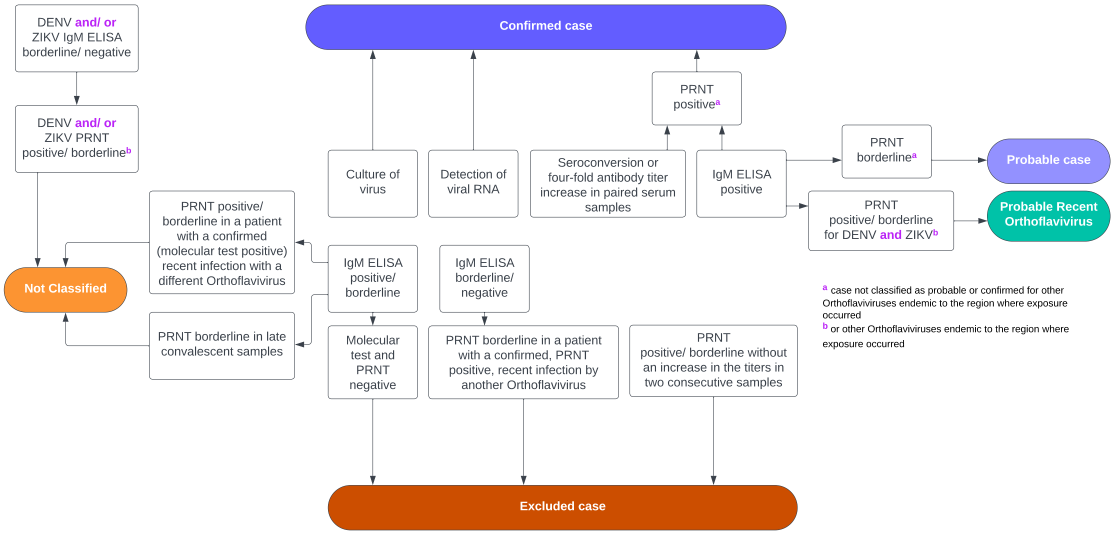

Figure S1: Flow chart of the criteria for the interpretation of laboratory tests results for Orthoflavivirus diagnosis (ZIKV/DENV) and case classifications.

Figure S2

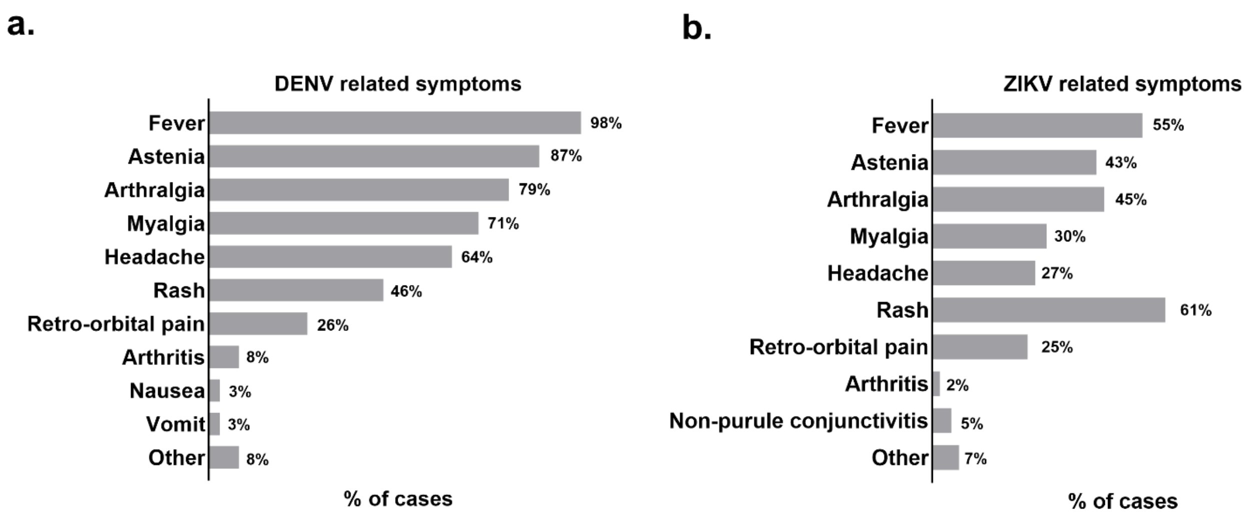

***Figure S2**: Symptomatology of DENV and ZIKV by % of confirmed and probable cases that data were available. a. Clinical symptoms of DENV confirmed and probable cases. b. Clinical symptoms of ZIKV confirmed cases.*

Figure S3

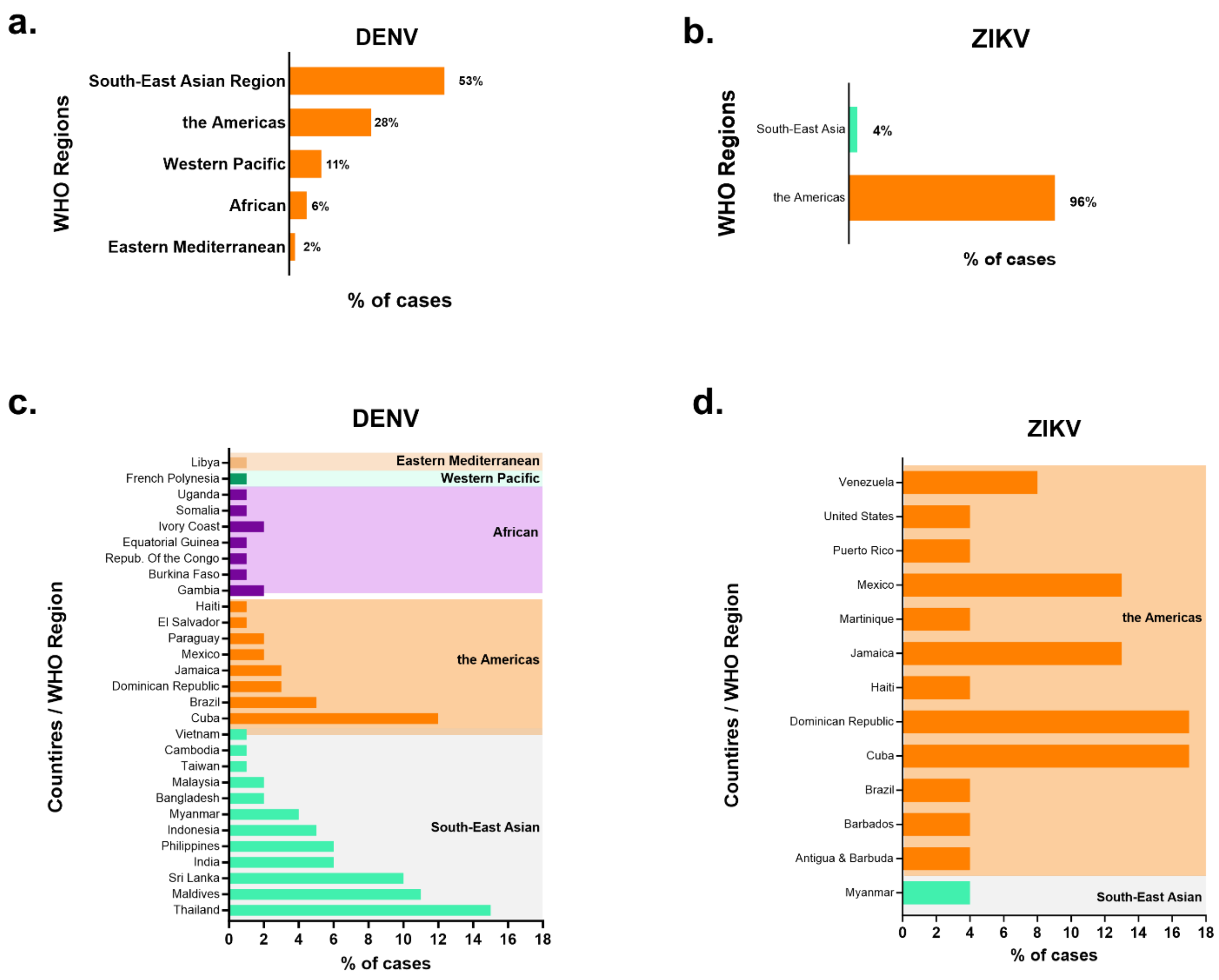

Figure S3: *Percentages of DENV and ZIKV confirmed and /probable cases by WHO region and by country visited before or during clinical symptoms presentation that data were available. Cases that had visited more than one country are not included in the graphs. a. Percentages of DENV confirmed and probable cases by WHO region. b. Percentages of ZIKV confirmed cases by WHO region. c. Percentages of DENV confirmed and probable cases by country. b. Percentages of ZIKV confirmed cases by country.*

Figure S4

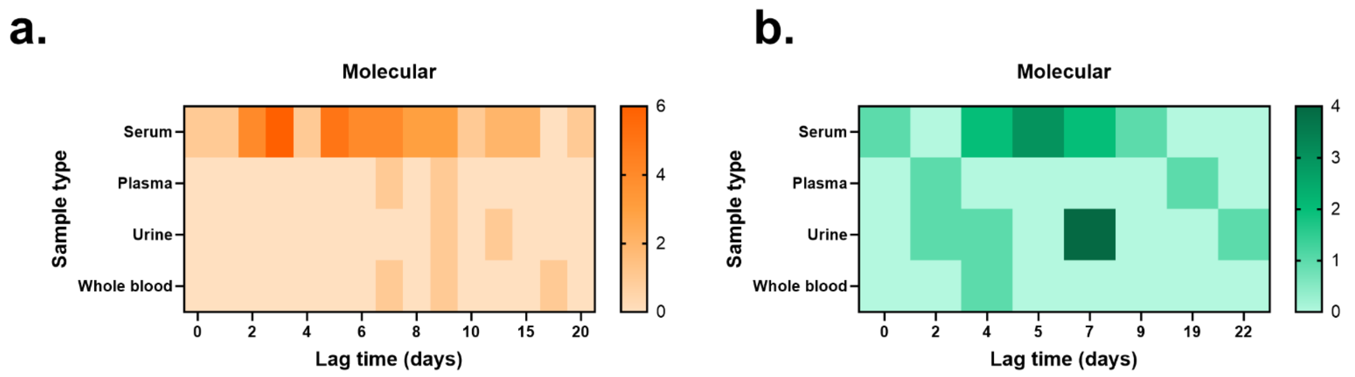

Figure S4: Heat map of sample type, no of samples per type and on lag time days for a. DENV and b. ZIKV that were tested by molecular assay.
